# Apolipoprotein C3 Polymorphisms, Cognitive Function and Diabetes in Caribbean Origin Hispanics

**DOI:** 10.1371/journal.pone.0005465

**Published:** 2009-05-08

**Authors:** Caren E. Smith, Katherine L. Tucker, Tammy M. Scott, Maria Van Rompay, Josiemer Mattei, Chao-Qiang Lai, Laurence D. Parnell, Mireia Junyent, Yu-Chi Lee, Bibiana Garcia-Bailo, José M. Ordovás

**Affiliations:** 1 Jean Mayer US Department of Agriculture Human Nutrition Research Center on Aging at Tufts University, Boston, Massachusetts, United States of America; 2 Tufts Medical Center, Tufts University, Boston, Massachusetts, United States of America; 3 Tufts University School of Nutrition, Boston, Massachusetts, United States of America; 4 University of Toronto, Toronto, Canada; Mayo Clinic College of Medicine, United States of America

## Abstract

**Background:**

Apolipoprotein C3 (APOC3) modulates triglyceride metabolism through inhibition of lipoprotein lipase, but is itself regulated by insulin, so that APOC3 represents a potential mechanism by which glucose metabolism may affect lipid metabolism. Unfavorable lipoprotein profiles and impaired glucose metabolism are linked to cognitive decline, and all three conditions may decrease lifespan. Associations between apolipoprotein C3 (*APOC3*) gene polymorphisms and impaired lipid and glucose metabolism are well-established, but potential connections between *APOC3* polymorphisms, cognitive decline and diabetes deserve further attention.

**Methods:**

We examined whether *APOC3* single nucleotide polymorphisms (SNPs) m482 (rs2854117) and 3u386 (rs5128) were related to cognitive measures, whether the associations between cognitive differences and genotype were related to metabolic differences, and how diabetes status affected these associations. Study subjects were Hispanics of Caribbean origin (n = 991, aged 45–74) living in the Boston metropolitan area.

**Results:**

Cognitive and metabolic measures differed substantially by type II diabetes status. In multivariate regression models, *APOC3* m482 AA subjects with diabetes exhibited lower executive function (*P* = 0.009), Stroop color naming score (*P* = 0.014) and Stroop color-word score (*P* = 0.022) compared to AG/GG subjects. *APOC3* m482 AA subjects with diabetes exhibited significantly higher glucose (*P* = 0.032) and total cholesterol (*P* = 0.028) compared to AG/GG subjects. *APOC3* 3u386 GC/GG subjects with diabetes exhibited significantly higher triglyceride (*P* = 0.004), total cholesterol (*P* = 0.003) and glucose (*P* = 0.016) compared to CC subjects.

**Conclusions:**

In summary, we identified significant associations between *APOC3* polymorphisms, impaired cognition and metabolic dysregulation in Caribbean Hispanics with diabetes. Further research investigating these relationships in other populations is warranted.

## Introduction

As individuals age, preserved cognitive function is associated with favorable lipid and glucose profiles, and these phenotypic traits often coexist in very long-lived individuals [1]–[3]. For example, plasma high density lipoprotein cholesterol (HDL-C) is positively correlated and triglycerides are negatively correlated with cognitive ability in centenarians [1], and unimpaired cognitive function is linked to reduced risk of death in elderly individuals [4]. Normal glucose metabolism, as evaluated by insulin sensitivity, is also characteristic of longer survival [3], [5], whereas diabetes increases mortality through increased cardiovascular or cerebrovascular disease risk [6], [7].

The favorable metabolic profiles and preserved cognitive function associated with healthy aging may be linked through common genotypes regulating lipoprotein metabolism such as cholesterol ester transfer protein [2]. Conversely, unfavorable metabolic profiles and cognitive decline which may be associated with vascular disease may be related through alleles such as *APOE* ε4 [8]. Another candidate gene that has been implicated in pathways influencing lipids, glucose metabolism and possibly cognition is *APOC3*, which encodes apolipoprotein C-III [3], [9].

APOC3 is a component of triglyceride-rich lipoproteins and is an important modulator of plasma triglycerides [10]. Inhibition of lipoprotein lipase by APOC3 and subsequent decreased triglyceride clearance may lead to hypertriglyceridemia [11]. Activation of endothelial cells and subsequent increased expression of adhesion molecules by APOC3 may represent additional mechanisms by which the protein contributes to atherogenesis [12]. Two of the most extensively studied variants of *APOC3*, 3u386 in the 3′ untranslated region, formerly known as SstI, and m482, a promoter single nucleotide polymorphism (SNP) located in an insulin response element (IRE), have been associated with alterations in both lipid and glucose metabolism [13]–[15]. *APOC3* m482 has been shown to act synergistically with variants of two other lipid regulators, *CETP* and *APOE*, to increase risk of coronary artery disease [16].

APOC3 also plays a role in diabetes, in which elevated plasma concentrations of both the apolipoprotein and triglycerides increase vascular disease risk [7]. Triglycerides were reported to be significantly higher for those with diabetes and carrying the variant allele for the m482 SNP [17]. Diabetes also increases the risk of cognitive impairment which is likely to be mediated, at least in part, through dysregulation of lipid metabolism and impaired vascular integrity [18]. However, with the exception of inconclusive reports evaluating relationships between *APOC3* and Alzheimer's disease [9], [19], the extent to which APOC3 is associated with cognitive impairment is largely unexplored. In the current study, we investigated associations between two *APOC3* SNPs (3u386 and m482) and cognitive function, and explored the metabolic measures which might underlie cognitive loss in a Caribbean Hispanic population. We investigated these relationships in the context of diabetes, which is characterized by metabolic dysregulation and is associated with cognitive loss.

## Methods

### Ethics Statement

All research involving human participants was approved by the authors' institutional review board or equivalent committee(s) and that board is named below. For research involving human participants, informed consent was obtained and all clinical investigation was conducted according to the principles expressed in the Declaration of Helsinki. The Institutional Review Board at Tufts University/New England Medical Center approved the protocol of the current study.

### Study Design and Subjects

Participants were recruited for a prospective two-year cohort study of men and woman of Puerto Rican origin, aged 45–75 years, and living in the Boston, Massachusetts metropolitan area. Data from the 2000 US Census were used to identify areas with a high density of Hispanic individuals in the target age range. Most participants were recruited through door-to-door enumeration. Blocks were visited at least three times and up to six times on different days of the week, including weekends, and at different times of the day. Participants were also identified by random approach at community festivals and other community events, through referrals from those invited to participate and through flyers distributed in the community. Eligibility requirements included self-reported Puerto Rican origin, the ability to answer interview questions in Spanish or English, living in the Boston area and being in the target age range. Individuals were excluded if serious health problems precluded answering interview questions, if the Mini Mental Status Examination Score (MMSE) was ≤10 or if they planned to move away from Boston within two years. Of 2004 individuals invited to participate, 1724 agreed to participate and 1357 completed the baseline interview. Individuals declined to participate due to lack of interest, lack of time or unwillingness to undergo blood draw. Genotyping was performed on 991 subjects, and analysis was limited to genotyped subjects.

Interviews to collect baseline demographic information, medical history and dietary data were conducted in the participants' homes between 2004 and 2008 by trained bilingual staff. Anthropometric data including height, weight, and waist circumference were measured in duplicate consistent with the technique used by the National Health and Nutrition Surveys. Blood was collected for biochemical analyses and genetic analysis, and was stored at −80°C.

### Genetic analysis

Genomic DNA was isolated from peripheral blood lymphocytes by standard methods. The single nucleotide polymorphisms (SNP) *APOC3* 3u386 (rs5128) and *APOC3* m482 (rs2854117), and (apolipoprotein E) *APOE*_C130R (rs429358) and *APOE*_R176C (rs7412) were genotyped using the ABI TaqMan SNP genotyping system 7900HT (Applied Biosystems, Foster City, California). Linkage disequilibrium (LD) between the two SNPs was estimated as a correlation coefficient (*r* ^2^) using Haploview software version 4.0 [20].

### Population ancestry admixture

The study subjects reflect three ancestral populations, European, Native American and African, which may be represented in varying proportions for each individual. One hundred ancestral markers were chosen to reduce confounding associated with population stratification [21]. The programs STRUCTURE 2.2 [22] and IAE3CI [23] were used to calculate three admixture values for each subject and achieved similar results [24]. Multivariate regression analysis models incorporate adjustment for population admixture through the addition of two of the three admixture variables as covariates.

### Cognitive Assessment and Factor Analysis

Cognitive ability was assessed using standard neuropsychological instruments administered in either Spanish or English, depending on the subject's preference.

Data reduction of cognitive test scores was conducted using principal components analysis to address issues associated with multiple testing. Interpretation of the resulting cognitive components was completed using Varimax rotation. Components with Eigen values greater than 1.0 were selected; cognitive tests that had factor loadings equal to or greater than 0.40 were considered to have primary loading on that factor. This analysis yielded three cognitive components interpreted as (1) executive function (planning, organization and multi-tasking), (2) memory, and (3) attention. Each cognitive component had a mean of 0.0 and standard deviation of 1.0. Composite variable were derived from specific tests as follows: executive function (Stroop color naming, Stroop word reading, Stroop color-word, verbal fluency, figure copying, clock drawing), memory function (word list learning immediate, word list learning delayed, word list learning percent retention, word list learning delayed recognition) and attention/concentration (verbal fluency, digit span forward, digit span backward and serial sevens subtraction from the MMSE).

### Statistical Analyses

All continuous variables were examined for normal distribution and a logarithmic transformation was applied for plasma triglycerides and glucose. The chi-square test was used to determine whether genotype distribution was consistent with Hardy-Weinberg equilibrium (HWE) expectations. Differences in cognitive function based on genotype were evaluated in the population in its entirety, using analysis of variance techniques. Differences in cognitive and metabolic measures between subjects with and without diabetes were evaluated using independent t-tests and subsequent genotypic analyses were performed in diabetics and non-diabetics separately. The relationships between *APOC3* genotypes and metabolic or cognitive measures were evaluated by analysis of variance techniques. For analyses involving genotype, three genotype groups were first considered to determine the most appropriate model. For *APOC3* m482 a recessive model was applied and for *APOC3* 3u386 a dominant model was applied. Multivariate regression models for cognitive measures included control for potential confounders including age, sex, education, income and ancestry admixture. Potential confounders for lipid and glucose measures included age, sex, alcohol (never, past, current), smoking (never, past, current), antilipemic medication, waist circumference and ancestry admixture. SAS (Version 9.1 for Windows) was used to analyze data. A *P* value of 0.05 was considered statistically significant.

## Results

Demographic, metabolic and genotypic data, divided by diabetes status, are presented in **Table 1** as unadjusted medians with the range of minimum and maximum values. Compared to those without diabetes, subjects with diabetes exhibited significantly lower executive function (*P*<0.0001), lower memory function (*P* = 0.038), lower attention/concentration function (*P* = 0.045), total cholesterol, HDL-C and low density lipoprotein cholesterol (LDL-C) (all *P*<0.0001), higher triglycerides (*P* = 0.0002), glucose, body mass index (BMI) and waist circumference (all *P*<0.0001) and older age (*P*<0.0001). Minor allele frequencies (MAF) of each SNP were 0.29 (*APOC3* 3u386) and 0.44 (*APOC3* m482) and frequencies did not differ significantly between subjects with and without diabetes. Genotype frequency for *APOC3* m482 did not deviate from HWE expectations but *APOC3* 3u386 frequency was not consistent with HWE. Evaluation of LD between the two SNPs revealed an r^2^ value of 0.024, indicating that SNPs were not in LD.

**Table 1:** Cognitive, metabolic and genotypic measures, by diabetic status.

	Non-diabetic (n = 583)	Diabetic(n = 408)	*P* value
Composite Cognitive Measures^1^			
Executive Function	0.262 (−3.22–2.88)	−0.201 (−2.83–2.19)	<.0001
Memory Function	0.119(−4.03–2.37)	0.029 (−4.04–2.40)	0.038
Attention/concentration	0.066(−3.31–3.17)	−0.040 (−3.57–3.17)	0.045
Demographic^1^			
Age(y)	56(45–75)	59(45–75)	<.0001
Female n (%)	418(71)	294(72)	0.943
Metabolic Measures^1^			
Total cholesterol (mmol/L)	4.9(2.3–9.7)	4.4(2.3–9.2)	<.0001
LDL-C (mmol/L)	3.0(0.8–5.5)	2.4(0.8–5.8)	<.0001
Triglyceride (mmol/L)	1.4(0.4–19.9)	1.6(0.4–14.5)	0.0002
HDL-C (mmol/L)	1.2(0.5–3.0)	1.1(0.5–2.2)	<.0001
Glucose(mmol/L)	5.4(3.8–6.9)	7.8(2.9–32.6)	<.0001
BMI (kg/m^2^)	30.2(17.0–59.9)	32.8(18.1–63.8)	<.0001
Waist (cm)	98(57–181)	105(67–167)	<.0001
Genotype			
*APOC3* m482			
GG	174(30.7)	132(34)	0.288
AG	275(48.6)	196(50)	
AA	117(20.7)	66(16)	
*APOC3* 3u386			
CC	318(57)	215(56)	0.614
GC	155(28)	102(26.6)	
GG	84(15)	67(17.4)	

1 Reported as unadjusted median (range).

Cognitive differences were first evaluated in the population in its entirety by *APOC3* 3u386 and *APOC3* m482 genotype, and no significant differences were found. Metabolic differences based on genotype were not evaluated in the population as a whole. Instead, based on the large number of highly significant cognitive and metabolic differences between subjects with and without diabetes (**Table 1**), we evaluated genotypic associations with phenotypic traits separately (diabetics and non-diabetics) for *APOC3* m482 (**Table 2**) and *APOC3* 3u386 (**Table 3**). Three genotype groups were first considered to determine the most appropriate model. For *APOC3* m482 a recessive model was applied and for *APOC3* 3u386 a dominant model was applied. Cognitive measures were evaluated by first testing for differences in overall functions (executive, memory and attention/concentration functions), and then continuing with appropriate individual test scores if differences were detected at the overall function level. For both SNPs, adjustment for ancestry admixture and for *APOE* ε4 carrier status were added as covariates to the models and these adjustments did not alter significant relationships. We observed a significant difference between *APOC3* m482 genotypes (GG, AG and AA) for executive function in those with diabetes (*P* = 0.021; data not shown) but cognitive associations were stronger when a recessive model was evaluated, and this model was used for all further comparisons for this SNP (Table 2). For those with diabetes but not for those without diabetes, executive function was lower in AA subjects (homozygous for the minor allele) compared to GG and AG subjects combined (*P* = 0.009). Three component tests, which were strongly correlated with executive function, were evaluated and component scores for two of these (Stroop color naming and Stroop color-word test) were lower in AA subjects (*P* = 0.014 for color naming and *P* = 0.022 for color-word). Total cholesterol (*P* = 0.028) and fasting glucose (*P* = 0.032) were higher in AA subjects. No significant cognitive or metabolic differences were observed for *APOC3* m482 genotypes in subjects without diabetes.

**Table 2:** Cognitive and metabolic measures for *APOC3* m482 in Caribbean Hispanics.

Diabetic	GG+AG(n = 328)	AA(n = 66)	*P* value
Cognitive Measures^1^			
Executive Function	−0.231±0.130	−0.559±0.167	0.009
Stroop color-naming	41.44±1.93	36.95±2.46	0.014
Stroop color-word	21.92±1.44	18.76±1.85	0.022
Stroop word	63.41±3.11	58.78±3.98	0.117
Memory Function	0.105±0.150	0.0761±0.193	0.844
Attention/concentration	0.150±0.151	0.141±0.194	0.951
Metabolic Measures			
Total cholesterol (mmol/L)^2^	4.40±0.07	4.74±0.14	0.028
LDL-C (mmol/L)^2^	2.44±0.06	2.65±0.12	0.093
Triglyceride (mmol/L)^2^	1.72±0.01	1.89±0.01	0.202
HDL-C (mmol/L)^2^	1.05±0.02	1.05±0.03	0.998
Glucose(mmol/L)^3^	8.12±0.06	9.08±0.06	0.032
BMI (kg/m^2^)^4^	32.54±0.45	34.13±0.89	0.094

Non-diabetic	GG+AG(n = 449)	AA(n = 117)	*P* value
Cognitive Measures^1^			
Executive Function	0.298±0.065	0.229±0.097	0.462
Stroop color-naming	47.54±0.96	45.95±1.42	0.244
Stroop color-word	25.78±0.73	25.34±1.08	0.671
Stroop word	73.52±1.50	71.74±2.23	0.408
Memory Function	0.116±0.073	0.210±0.108	0.369
Attention/concentration	0.217±0.075	0.368±0.111	0.163
Metabolic Measures			
Total cholesterol (mmol/L)^2^	4.80±0.06	4.73±0.10	0.509
LDL-C (mmol/L)^2^	2.83±0.05	2.76±0.09	0.479
Triglyceride (mmol/L)^2^	1.61±0.01	1.60±0.01	0.874
HDL-C (mmol/L)^2^	1.14±0.02	1.13±0.03	0.893
Glucose(mmol/L)^3^	5.43±0.06	5.43±0.06	0.908
BMI(kg/m^2^)^4^	30.1±0.32	29.9±0.59	0.840

Data are reported as mean±standard error of the mean.

1 Adjusted for age, gender, admixture, education and income.

2 Adjusted for age, gender, admixture, smoking, drinking, waist circumference, antilipemic medication.

3 Adjusted for age, gender, admixture, smoking, drinking, waist circumference.

4 Adjusted for age, gender, admixture, smoking, drinking.

**Table 3:** Cognitive and metabolic measures for *APOC3* 3u386 in Caribbean Hispanics.

Diabetic	CC(n = 215)	GC+GG(n = 169)	*P* value
Cognitive Measures^1^			
Executive Function	−0.262±0.140	−0.282±0.142	0.847
Memory Function	0.080±0.159	0.136±0.161	0.629
Attention/concentration	0.157±0.159	0.111±0.162	0.694
Metabolic Measures			
Total cholesterol (mmol/L)^2^	4.29±0.09	4.67±0.10	0.003
LDL-C (mmol/L)^2^	2.40±0.07	2.58±0.03	0.079
Triglyceride (mmol/L)^2^	1.62±0.01	1.91±0.01	0.004
HDL-C (mmol/L)^2^	1.06±0.02	1.05±0.02	0.865
Glucose(mmol/L)^3^	7.91±0.06	8.74±0.06	0.016
BMI (kg/m^2^)^4^	32.3±0.55	33.4±0.60	0.161

Non-diabetic	CC(n = 318)	GC+GG(n = 239)	*P* value
Cognitive Measures^1^			
Executive Function	0.308±0.070	0.253±0.082	0.508
Memory Function	0.161±0.079	0.071±0.092	0.335
Attention/concentration	0.261±0.080	0.260±0.093	0.992
Metabolic Measures			
Total cholesterol (mmol/L)^2^	4.76±0.07	4.84±0.08	0.352
LDL-C (mmol/L)^2^	2.78±0.06	2.86±0.07	0.348
Triglyceride (mmol/L)^2^	1.57±0.01	1.68±0.01	0.184
HDL-C (mmol/L)^2^	1.15±0.02	1.12±0.03	0.334
Glucose(mmol/L)^3^	5.42±0.06	5.47±0.06	0.357
BMI(kg/m^2^)^4^	29.5±0.38	30.7±0.43	0.035

Data are reported as mean±standard error of the mean.

1 Adjusted for age, gender, admixture, education and income.

2 Adjusted for age, gender, admixture, smoking, drinking, waist circumference, antilipemic medication.

3 Adjusted for age, gender, admixture, smoking, drinking, waist circumference.

4 Adjusted for age, gender, admixture, smoking, drinking.

For *APOC3* 3u386, associations were also evaluated separately in subjects with and without diabetes (Table 3). No significant differences between genotypes were observed for any of the overall cognitive measures in either group and no individual test scores were evaluated. For metabolic measures for *APOC3* 3u386, differences were most apparent in the context of a dominant model, which was used for all evaluations. In subjects with diabetes, total cholesterol (*P* = 0.003), triglycerides (P = 0.004) and fasting glucose (*P* = 0.016) were significantly higher in carriers of the variant allele (GC and GG) compared to non-carriers (CC). In subjects without diabetes, only BMI was higher (*P* = 0.035) in GC and GG subjects compared to CC.

## Discussion

We observed significant associations for two *APOC3* SNPs, m482 and 3u386, with cognitive and metabolic measures in subjects with diabetes in a Caribbean Hispanic population. For *APOC3* m482, those with diabetes and the AA genotype exhibited reduced cognitive function, as shown by lower executive function and lower component test scores (Stroop tests). In contrast, for *APOC3* 3u386, cognitive function did not vary by genotype, but minor allele carriers (GC and GG) with diabetes exhibited higher plasma triglycerides. For both SNPs, the minor allele in subjects with diabetes was associated with less favorable cholesterol and glucose measures, compared to those with diabetes who lacked the minor allele (G) for the 3u386 SNP and compared to non-homozygous minor subjects (GG and AG) for the m482 SNP.

Previous studies specifically examining associations between *APOC3* genetic variants and cognition are limited and inconclusive. One early study was negative for an association between the *APOC3* 3u386 SNP and familial Alzheimer's disease in a United Kingdom population [19], and a second more recent study reported a weak, protective association of the minor allele of the 3u386 SNP for sporadic Alzheimer's disease in a Chinese population [9]. Neither of these studies reported metabolic measures for their subjects.

Despite limited evidence for associations between *APOC3* genotype and cognition, linkages between *APOC3* SNPs and the metabolic dysregulation which may accompany cognitive impairment in older individuals provide potential mechanistic explanations for the associations we observed. Variants of *APOC3* 3u386 were associated with alterations in lipids, including increased total cholesterol and triglycerides, and in markers of glucose metabolism [13], [14]. Variants of *APOC3* m455 and m482 have been associated with altered glucose metabolism and metabolic syndrome [15], [25]. A fourth *APOC3* SNP, m641, has been associated with improved insulin sensitivity and favorable lipoprotein profiles in Ashkenazi centenarians and their offspring [3].

Results from the current study are consistent with studies cited above which reported associations of *APOC3* with altered glucose metabolism. Earlier studies have also reported associations between diabetes and impaired cognition [18], an association which is apparent in the current study as well. For subjects with diabetes in the current study, all three overall cognitive function scores (executive, memory and attention/concentration) were significantly lower compared to those without diabetes, independently of *APOC3* genotype. Within the subset of subjects with diabetes, lower cognitive function was associated with m482 and the variant allele of this SNP was also associated with increased fasting glucose. The IRE location of m482 suggests that this SNP could render control of transcription of *APOC3* sensitive to changes in glucose metabolism. *APOC3* transcription has been shown to be down-regulated by insulin, but reports differ regarding the role of the IRE in mediating this process. Genetic variants of m482 have been shown to be associated with loss of insulin downregulation of *APOC3* transcription [26], but in a population with familial combined hyperlipidemia, insulin regulation of transcription was shown to be independent of the IRE-based variants [27]. Subsequent animal studies provided evidence that insulin inhibition of APOC3 is mediated by nuclear transcription factor forkhead box (Foxo1) which targets the *APOC3* promoter. Elevated Foxo1, which occurs under conditions of both insulin absence (type 1 diabetes) and insulin resistance (type 2 diabetes), is associated with increased triglyceride production in mice [28]. Dietary factors (including energy and protein intake) which modify Foxo1 post-translationally in animals are potential sources of additional complexity in *APOC3* regulation [29]. Further, in the current study population, the chronic hyperglycemia and hyperinsulinemia associated with type 2 diabetes could impair normal physiologic regulation.

Altered lipid metabolism, like altered glucose metabolism, has been associated both with APOC3 concentration and also with altered cognitive ability, although all three variables were not previously shown to be related. Plasma triglycerides are negatively correlated with cognitive function in very old individuals [1] and reducing triglycerides with antilipemic drugs has been shown to improve cognitive performance [30]. In the current study, associations with triglycerides were observed for *APOC3* 3u386 alone, although both 3u386 and m482 were associated with increased total cholesterol. Increased total cholesterol has been shown to be a risk factor for dementia/Alzheimer's disease and also for milder forms of cognitive impairment, particularly if cholesterol concentrations are high at mid-life [31].

Both the associations reported for the current study and those described for other populations depend, in part, on the allelic frequencies of the *APOC3* SNPs we examined, which are highly variable across ethnic groups. For *APOC3* m482, the MAF has been reported as 0.25 in Whites [of European ancestry], 0.71 in Africans [of Yoruban ancestry] and 0.44 in South Asians [25]. In the current study of Caribbean Hispanics, MAF was 0.44. For *APOC3* 3u386, MAF has been reported as 0.17 in African-Americans and 0.095 in Whites [32] but as high as 0.42 in a hypertriglyceridemic Chinese population [34]. In the current population, *APOC3* 3u386 MAF was 0.29. Genetic associations are also modulated by genetic background, including that of other SNPs in the *APOA1/APOC3/APOA4/APOA5* gene cluster. For example, the association of *APOC3* 3u386 with triglycerides was shown to be not independent of associations of triglycerides with *APOA5* [32] and we cannot eliminate the possibility of similar relationships in our study population.

The deviation from HWE for the *APOC3* 3u386 SNP requires some discussion, because evaluation of HWE has been used as a tool to detect genotyping error. Genotyping error rates are very low with the methods we used. Further, HWE deviation may also reflect population substructure or selection, and may be interpreted as supportive of an association between genotype and disease [34]. Results from the genetic neutrality test support the hypothesis that differences in *APOC3* allele frequencies among populations reflect selective pressures on the *APOA1/APOC3* locus [10], [35]. Departure from HWE for the *APOC3* 3u386 SNP has been described for other populations, including a sample composed of French-Canadians and non-French Canadians, in which the departure appears to be related to different genetic structures in the two sub-groups [36]. As population stratification is also an important aspect to the analysis of genetic data of Caribbean Hispanics, we have adjusted by admixture in our association testing to reduce confounding associated with population sub-structure. Deviation from HWE for this SNP was also documented for a Korean population comparing hypertriglyceridemic and control subjects in which the deviation was due to a low frequency of homozygosity for the risk allele in control subjects [37]. Within the *APOA1/APOC3/APOA4/APOA5* cluster, deviations from HWE are not always limited to *APOC3* 3u386 because other deviations have been reported close to the 3′UTR region in which this SNP is located. In a study investigating twelve *APOA1/APOC3* SNPs in two populations, significant HWE deviations were present for three SNPs in African-Americans and five SNPs in European-Americans [38]. Collectively, these results are evidence of alternative explanations for the observed HWE deviation in our population.

In summary, our results support an association between *APOC3* genotype and cognition in people with diabetes, which may be mediated through established vascular disease risk factors. Variant alleles of both *APOC3* 3u386 and *APOC3* m482 were associated with unfavorable lipid and glucose markers which contribute to cardiovascular and cerebrovascular disease risk. *APOC3* m482 was also associated with decreased cognitive function. While links between *APOC3* and metabolic dysregulation have been extensively documented, and favorable lipid profiles may be associated with cognitive health, the observed combined associations between *APOC3* genotype, altered glucose and lipid metabolism and cognitive function has not been previously reported. Further research in additional populations, particularly those with a high prevalence of diabetes, will be needed in order to confirm these preliminary findings.
